# Membrane Transporters and Pharmacological Implications

**DOI:** 10.4172/2329-6887.1000e155

**Published:** 2016-06-08

**Authors:** Luca Cucullo, Taylor Liles

**Affiliations:** 1 Department of Pharmaceutical Sciences, 1300 S Coulter St, Amarillo TX 79106-1712, USA; 2 Center for Blood-Brain Barrier Research, Texas Tech University Health Sciences Center, Amarillo, TX 79106, USA

## Introduction

Membrane transporters serve to move chemicals in and out of the cells according to metabolic needs or the presence of toxic compounds. These processes are mediated by facilitated diffusion or active transport through the lipid bilayer that is the cell membrane. There exist two main categories of membrane transporters, the more passive solute carrier transporters (SLCs) and the ATP binding cassette transporters (ABCs). SLCs allow for passage of ions, sugars, lipids, amino acids and other compounds down a gradient, contributing to a cell’s passive permeability for such compounds. ABCs feature a highly conserved nucleotide binding domain (NBD) which contain peptide sequences responsible for ATP hydrolysis such as the Walker A and B motifs. ABCs utilize the energy stored in ATP to transport chemicals against their concentration and/or electrical gradient and consequently alter expected biological conditions. Transporters are now recognized as crucial barriers (e.g., efflux transporters) as well as possible delivery pathways to consider when designing new pharmaceutical agents as many traditional therapeutics are being recognized as transporter substrates [1–3]. Drug resistant tumors and the blood-brain barrier (BBB) for example have been shown to actively express efflux transporters preventing therapeutic agents from reaching clinically relevant intracellular concentrations and/or physiological targets in the brain [4–7].

Consequently, in 2010 the International Transporter Consortium put forth the ‘white paper’ detailing the structure, location, and known substrates of various pharmacologically relevant transporters. This prompted action from American and European regulatory agencies to release guidelines on transporter-drug interaction studies [3]. P-Glycoprotein (MDR1), Breast Cancer Resistance Protein (BCRP), Organic Anion Transporter peptide (OATP), Organic Anion Transporter (OAT), Organic Cation Transporter (OCT), and Multi Drug Resistance Protein (MRP) are mentioned directly in the white paper and have been a focus of the ITC to be included in the in vitro to in vivo extrapolation (IVIVE) which refers to the qualitative or quantitative transposition of in vitro experimental results to predicts a physiological and/or pathological phenomena in vivo. P-glycoproteins (Pgp) and the Cytochrome P450 enzyme CYP3A analogs share a significant number of substrates [8] and are both found in the intestines and liver [9–11]. Together they compose a first pass metabolism barrier for therapeutic agents taken orally [12,13]. Because Pgp effluxes compounds from the intestinal wall back into the lumen of the intestines, researchers may mistake increased mean residence time (MRT) for increased absorption when performing PK/PD studies [14]. Some of these CYP analogs such as CYP3A4, CYP2C9, CYP2C19, CYP2A6 and CYP2E1 are also expressed at the BBB endothelial level of the BBB under pathological conditions (e.g., drug resistant epilepsy). In some cases, the expression of a CYP enzyme (e.g., CYP3A CYP2C19 and CYP2C9) is regulated by the activation of the xenobiotic receptor pregnane X receptor (PXR) which also controls the expression of Pgp and other drug efflux systems [15,16]. With regard to the drug efflux transporters at the BBB level, multidrug resistance (MDR) is a major obstacle to treating patients with cancer and is often the result of overexpression of a 170- to 180-kDa plasma membrane glycoprotein known as P-glycoprotein (Pgp) [17,18] and multidrug resistance-related proteins (MRP1, 190 kDa) [19,20]. Human Pgp is encoded by MDR1 and rodent Pgp by Mdr1a and Mdr1b [21–24]. Pgp and MRPs belong to the superfamily of ATP-binding cassette transporters. Unlike other selective (classical) transport proteins, MDR proteins recognize a wide range of substrates. This wide substrate specificity explains the cross-resistance to several chemically unrelated compounds, the characteristic feature found in the multi-drug resistance phenotype. In addition to their overlapping substrates specificity, each transporter can handle unique compounds. Pgp-MDR1 is a transporter for large amphipathic compounds either uncharged or slightly charged while the MRP family is mostly transporting hydrophobic anionic conjugates with glucuronide, sulfate or glutathione and also extrudes hydrophobic uncharged drugs [19]. Experiments designed to define the structure of Pgp suggest that there is no simple single drug-binding site or pore in Pgp. Amino acid substitutions in, or near, most of the transmembrane segments affect substrate specificity efficiency or transport. In drug refractory patients a synergistic effect between MDRs and CYP enzymes has been recently observed which antagonizes the passage of drugs targeting the brain (e.g., antiepileptics, tumor suppressants, etc.) through a concerted set of mechanisms [15]. The efflux transporters extrude the drug from the brain across the BBB back into the blood circulation while the CYP enzyme metabolize the drug substrate into (at large) inactive derivate(s), thus rendering the drug bioavailability ineffective from a therapeutic standpoint. Other than MDR, altered activity of efflux transporters have been linked to a number of neurological disorders including Alzheimer’s disease [25], Parkinson’s diseases [26] and Creutzfeldt-Jakob disease [27].
